# Improvement of Post-inflammatory Hyperpigmentation in a Second-Degree Burn Scar Following the NewMelan Depigmentation Protocol: A Case Report

**DOI:** 10.7759/cureus.114890

**Published:** 2026-08-20

**Authors:** Maribel C Sanchez Meza

**Affiliations:** 1 Plastic Surgery, Sculpt Clinic, Caracas, VEN

**Keywords:** burn scar, compression garment, depigmentation therapy, epidermal turnover, friction burn, hyperpigmented scar, melanogenesis, newmelan, post-inflammatory hyperpigmentation, tyrosinase inhibition

## Abstract

Post-inflammatory hyperpigmentation is a common sequela of second-degree burns and may result in persistent discoloration after complete wound healing. This case report describes a 65-year-old woman who developed persistent hyperpigmentation of the abdominal skin following a friction-induced second-degree burn associated with prolonged use of a compression garment. Despite complete wound healing and subsequent abdominoplasty, residual hyperpigmentation persisted. A more specific classification of the original burn as superficial or deep partial-thickness was not documented at the time of injury.

Clinical examination revealed hyperchromia and uneven pigmentation localized to the previously injured area, clinically consistent with post-inflammatory hyperpigmentation. The diagnosis was based on the clinical history and examination; no dermoscopy, Wood's lamp examination, biopsy, or other confirmatory diagnostic assessment was performed. The patient was treated using the NewMelan depigmentation protocol, a multimodal approach intended to target pathways involved in melanogenesis and epidermal turnover.

At approximately one month of follow-up, a visually apparent reduction in pigmentation intensity and improvement in skin tone uniformity were observed on clinical examination and serial photographs. No objective pigmentation measurement or validated scar assessment scale was used, and the photographs were not obtained using a fully standardized photographic protocol. No treatment-related adverse effects or complications were reported during the documented one-month observation period.

This case describes clinical improvement in post-inflammatory hyperpigmentation observed following the use of a multimodal depigmentation protocol. Because this is a single uncontrolled case with subjective outcome assessment and short-term follow-up, treatment efficacy, causality, and long-term safety cannot be established. Further studies using objective pigmentation measurements, standardized photographic assessment, comparator groups, and longer follow-up are needed.

## Introduction

Post-inflammatory hyperpigmentation is a common sequela of cutaneous injury and may occur following second-degree burns [1-4]. Residual hyperpigmentation and dyschromia may persist after wound healing, resulting in clinically significant pigmentary changes and aesthetic concerns [5,6]. The development and persistence of post-inflammatory hyperpigmentation can be influenced by multiple factors, including the degree of inflammation, characteristics of the initial injury, ultraviolet exposure, and individual pigmentation characteristics [7,8].

Management of post-burn hyperpigmentation remains challenging because pigmentary alterations may persist after wound closure [9,10]. Reported management approaches include photoprotection, topical depigmenting agents, chemical exfoliation, and selected laser or light-based therapies [11-15]. However, evidence specifically addressing post-burn hyperpigmentation is more limited than the broader literature concerning post-inflammatory hyperpigmentation from other causes, and treatment response may vary among patients.

The NewMelan protocol is a proprietary multimodal depigmentation protocol that has previously been described in a case involving melasma and lentigines [16]. Although individual depigmenting agents used in multimodal approaches may target pathways involved in melanogenesis and epidermal turnover, evidence regarding the use of the NewMelan protocol specifically for post-burn post-inflammatory hyperpigmentation is limited. The previously published NewMelan case involved facial melasma and lentigines rather than burn-related pigmentation and therefore does not establish efficacy for post-burn hyperpigmentation. Accordingly, the use of this protocol in the present case is reported as a clinical observation rather than as evidence of superiority over established treatment approaches or proven efficacy for post-burn hyperpigmentation. Skin phototype may be characterized using the Fitzpatrick skin classification system [17]; however, a definitive Fitzpatrick phototype was not documented for the patient in the present case.

This case report describes the clinical course of a 65-year-old woman who developed persistent post-inflammatory hyperpigmentation of the abdominal skin following a friction-induced second-degree burn associated with prolonged use of a compression garment. The clinical interest of this case lies in the use of the NewMelan depigmentation protocol in persistent pigmentation following a burn, a clinical context distinct from the previously reported application for melasma and lentigines. The report describes the observed clinical course following treatment while recognizing that, as a single uncontrolled case, it cannot establish treatment efficacy or causality.

## Case presentation

**Figure 1:**
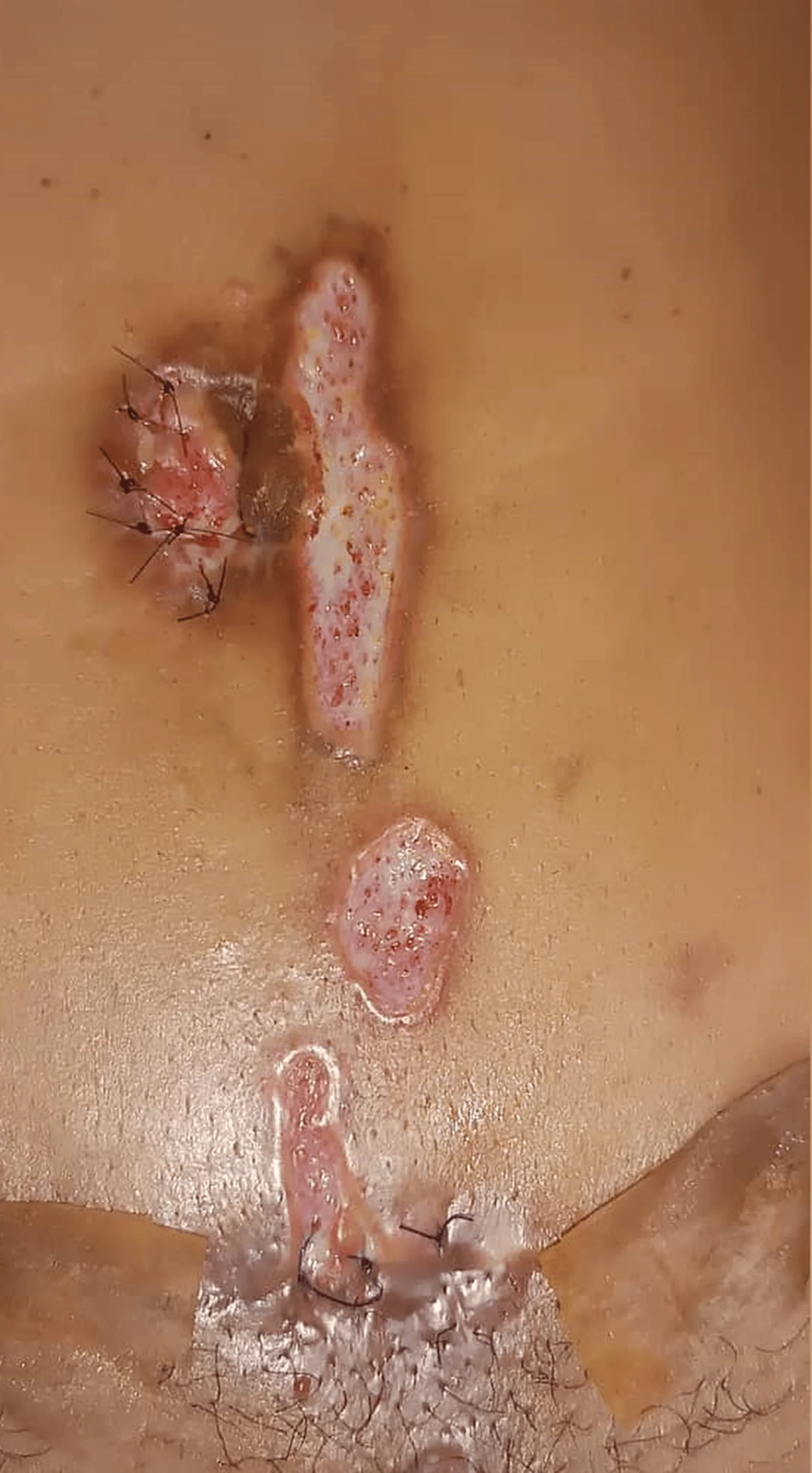
Abdominal skin injury associated with a friction-induced second-degree burn. Clinical photograph documenting the abdominal skin injury associated with prolonged use of a compression garment. Areas of epidermal loss and healing tissue are visible. Sutures visible in the photograph are associated with the subsequent abdominoplasty. The precise timing of the photograph relative to the initial injury and surgical procedure could not be established from the available documentation.

A 65-year-old woman presented with persistent hyperpigmentation of the abdominal skin following a friction-induced second-degree burn associated with prolonged use of a compression garment. According to the patient, continuous friction and pressure from the garment resulted in blister formation and subsequent skin injury (Figure 1).

The available clinical documentation did not specify whether the original injury was superficial or deep partial thickness; therefore, a more specific burn depth classification could not be assigned retrospectively.

The patient subsequently underwent a classic abdominoplasty in an attempt to improve the appearance of the affected abdominal area; however, residual pigmentation persisted. The available documentation did not establish the precise interval between the burn and abdominoplasty or whether the previously affected skin was excised or preserved during surgery. Therefore, a potential contribution of the surgical procedure to the subsequent pigmentation could not be excluded. After approximately six months of complete wound healing, a persistent area of dark discoloration remained, causing aesthetic concern and prompting the patient to seek treatment.

Clinical examination revealed hyperchromia and uneven pigmentation extending from the umbilical region into the lower abdomen and localized to the previously affected area. No active inflammation, infection, ulceration, or drainage was present at the time of evaluation. Exact lesion dimensions and objective measurements of pigmentation or scar characteristics were not documented, and no validated pigmentation or scar assessment scale was used. The diagnosis of post-inflammatory hyperpigmentation was based on the clinical history and examination. No dermoscopy, Wood's lamp examination, biopsy, histopathologic examination, or other confirmatory diagnostic assessment was performed; therefore, alternative causes of persistent pigmentation were not objectively excluded. A single definitive Fitzpatrick skin phototype was not documented in the original clinical record; therefore, no specific Fitzpatrick phototype was retrospectively assigned.

A depigmentation regimen using the NewMelan protocol was initiated. NewMelan was used as a proprietary multimodal depigmentation protocol. The available clinical documentation did not contain sufficient information to report the exact concentrations of all active ingredients, vehicle, pH, quantity applied per application, or other complete formulation details; therefore, these details could not be accurately reconstructed retrospectively. The patient was instructed to follow the prescribed application schedule; however, sufficiently detailed documentation regarding concomitant skincare, photoprotection, and adherence assessment was not available.

**Figure 2:**
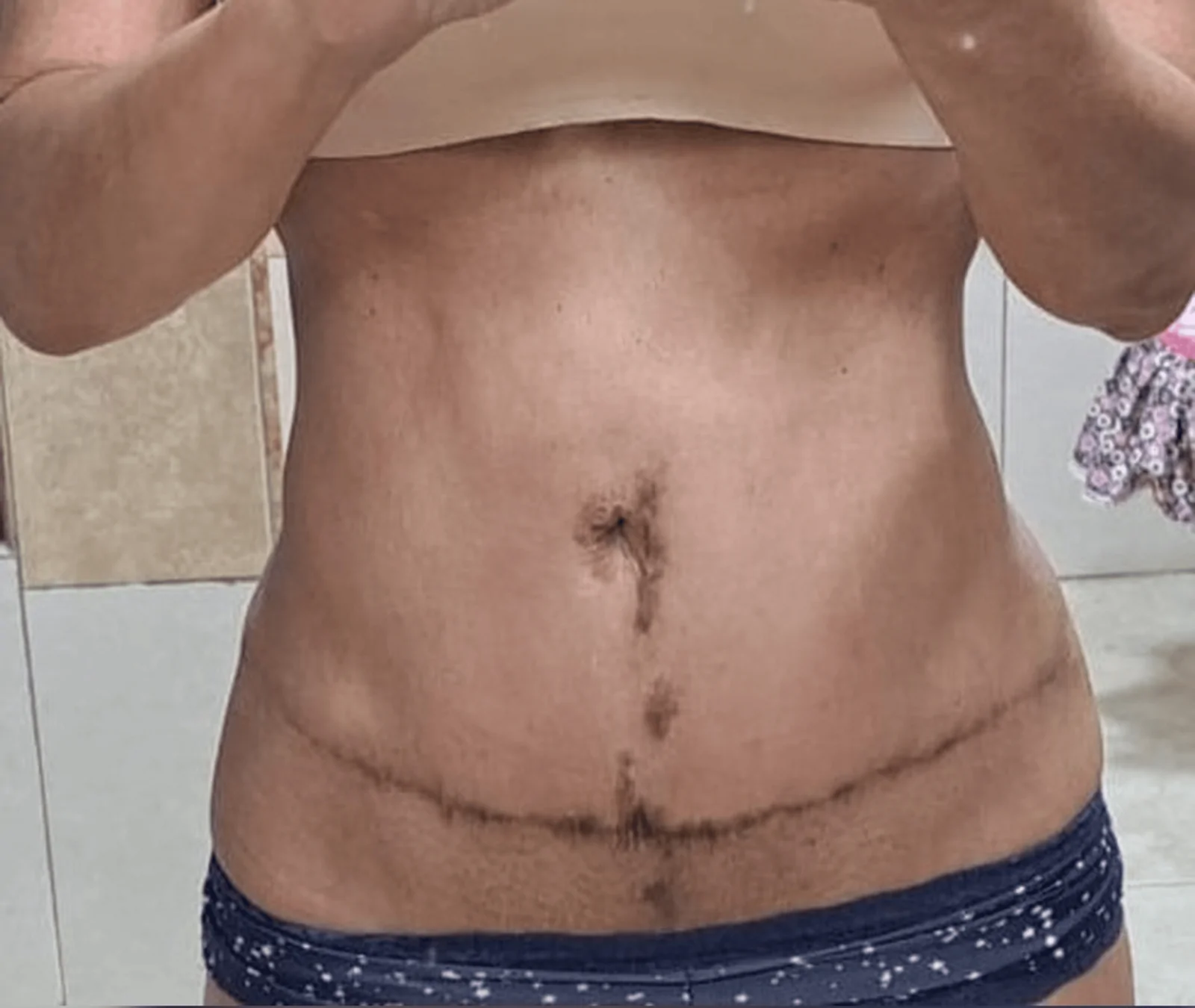
Baseline appearance of post inflammatory hyperpigmentation before treatment. Clinical photograph obtained after complete wound healing and before initiation of the NewMelan depigmentation protocol, demonstrating residual hyperchromia and uneven pigmentation extending from the umbilical region into the lower abdomen. No objective pigmentation measurement, scale marker, or standardized digital image analysis was used. Black squares were digitally added only where necessary to obscure potentially identifying marks or characteristics and protect patient confidentiality in accordance with editorial guidance; they do not represent tattoos or treatment markings.

Baseline clinical photographs were obtained before initiation of therapy (Figure 2).

**Figure 3:**
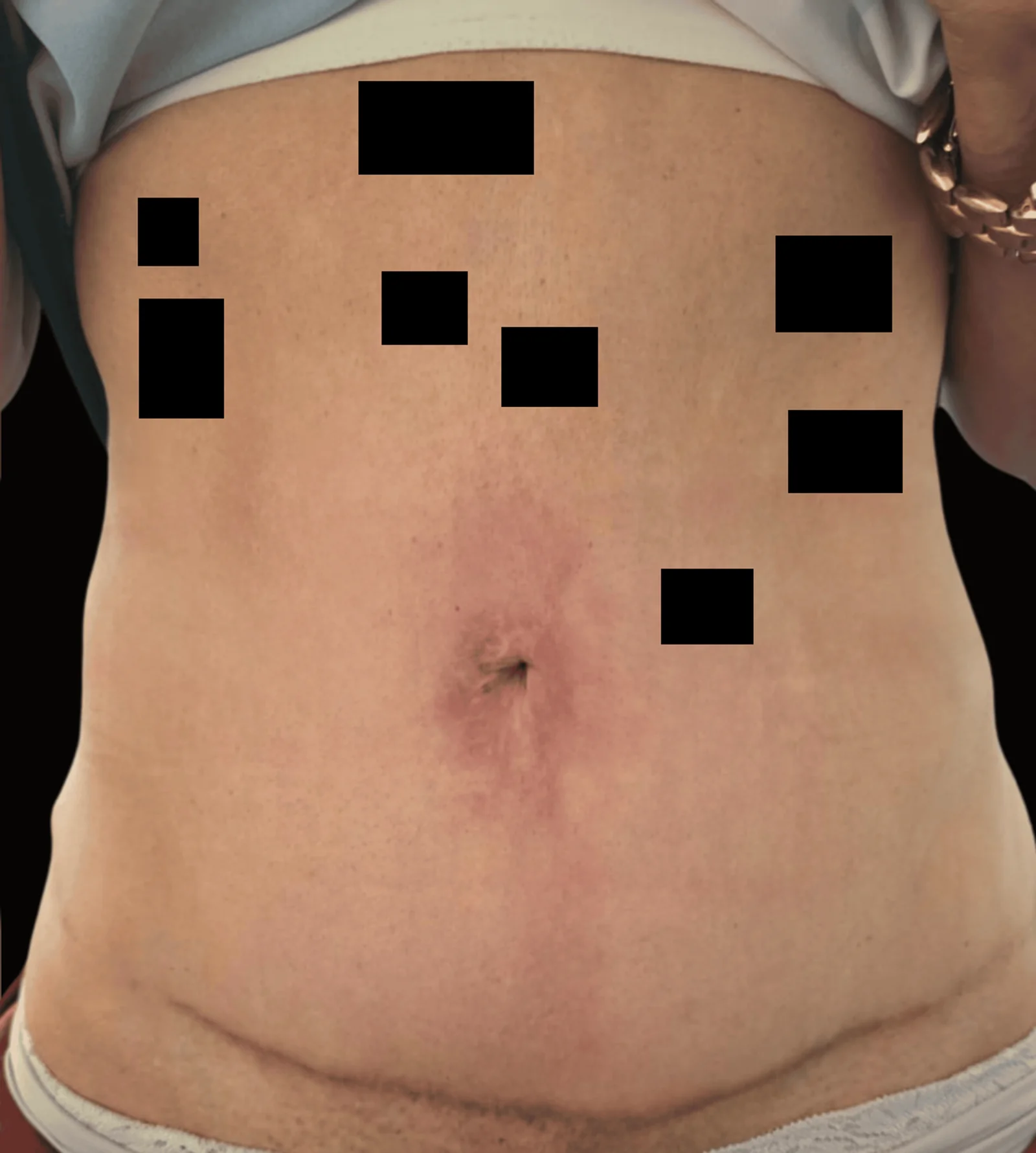
Appearance one month after initiation of the NewMelan depigmentation protocol. Clinical photograph obtained approximately one month after initiation of the NewMelan depigmentation protocol. Visual comparison with the baseline photograph suggests reduced pigmentation intensity and improved skin-tone uniformity. No objective pigmentation measurement or validated scar assessment was performed, and fully standardized photographic conditions were not documented. Therefore, the photograph represents a descriptive clinical observation and does not independently establish treatment efficacy.

At approximately one month of follow-up, a visually apparent reduction in pigmentation intensity and improvement in skin tone uniformity were observed on clinical examination and serial photographic assessment (Figure 3).

No objective pigmentation measurement, standardized digital image analysis, validated pigmentation scale, or validated scar assessment scale was used. The available records also did not establish that the baseline and follow-up photographs were obtained using identical lighting, camera settings, distance, positioning, exposure, or white balance. Therefore, the observed changes represent descriptive clinical observations rather than objectively measured treatment outcomes, and photographic variability may have influenced the apparent degree of change.

During the documented one-month observation period, no treatment-related adverse effects or complications were reported by the patient. A formal validated adverse event assessment instrument was not used, and the short follow-up period does not permit conclusions regarding delayed adverse reactions, recurrence, rebound hyperpigmentation, hypopigmentation, or long-term tolerability.

Written informed consent was obtained from the patient for treatment and publication of clinical information and photographs in an Open Access journal.

## Discussion

Post-inflammatory hyperpigmentation is a common consequence of cutaneous injury and may develop following inflammatory conditions, trauma, burns, and other forms of skin damage [1-4]. The condition is associated with increased melanocyte activity and melanin deposition within the epidermis and, in some cases, the dermis. The development and persistence of post-inflammatory hyperpigmentation may vary according to the characteristics of the initial injury, degree and duration of inflammation, ultraviolet exposure, individual pigmentation characteristics, and other patient-related factors [5-8].

Second-degree burns may result in persistent pigmentary alterations after complete wound closure. Residual dyschromia and hyperchromia may persist for months or longer and can become an important aesthetic concern. The severity and duration of post-burn hyperpigmentation may be influenced by burn depth, duration of inflammation, ultraviolet exposure, and individual healing characteristics [9,10]. Importantly, post-inflammatory hyperpigmentation may also fade spontaneously over time. Therefore, in an uncontrolled case such as the present report, the degree to which the observed pigmentary change represents the natural evolution of the condition rather than an effect of treatment cannot be determined.

Management approaches for post-inflammatory and post-burn hyperpigmentation include photoprotection and topical depigmenting agents, such as hydroquinone, azelaic acid, retinoid-containing regimens, and combination therapies. Chemical peels and selected laser or light-based interventions have also been described, although treatment response and the risk of treatment-associated pigmentary alteration may vary among patients [11-15]. Evidence supporting these approaches varies, and evidence derived from post-inflammatory hyperpigmentation of different etiologies should not necessarily be interpreted as evidence specific to post-burn hyperpigmentation. The present case was not designed to compare NewMelan with hydroquinone, combination topical therapy, chemical peels, laser-based treatment, microneedling, or other established approaches; therefore, no conclusions regarding comparative effectiveness or superiority can be made.

NewMelan was used in this case as a proprietary multimodal depigmentation protocol. A previously published case report described use of the NewMelan multimodal depigmentation protocol in a patient with facial melasma and lentigines [16]. However, that report involved a different pigmentary disorder and does not establish efficacy of NewMelan for post burn post inflammatory hyperpigmentation. The available clinical documentation in the present case did not contain sufficiently complete information regarding the exact concentrations of all active ingredients, vehicle, pH, quantity applied, or whether all reported components were present simultaneously within a single formulation or administered as separate components. Accordingly, these formulation characteristics cannot be accurately reconstructed retrospectively, which limits reproducibility and interpretation of the intervention. Although individual depigmenting agents have been reported to affect pathways involved in melanogenesis and epidermal turnover, evidence concerning the biological activity of individual ingredients does not establish that the proprietary combination caused the clinical changes observed in this patient.

Skin phototype can be characterized using the Fitzpatrick skin classification system [17]. However, a single definitive Fitzpatrick phototype was not documented in the original clinical record for the patient in this case. Therefore, the previously reported classification of Fitzpatrick skin type III-IV was removed, and a specific phototype was not retrospectively assigned.

In the present case, a visually apparent reduction in pigmentation intensity and improvement in skin tone uniformity were observed approximately one month after initiation of treatment. These findings were based on clinical examination and serial photographic assessment rather than objective quantitative measurement. No colorimetry, melanin index, validated pigmentation scale, standardized digital image analysis, or blinded independent assessment was performed. Furthermore, the available records do not establish that baseline and follow-up photographs were obtained under identical lighting, camera settings, exposure, white balance, distance, or positioning. Consequently, observer bias and photographic variability may have influenced the apparent degree of change.

The temporal association between treatment and the observed clinical change should not be interpreted as evidence of causality. There was no untreated control or comparator, and the natural course of post-inflammatory hyperpigmentation, including spontaneous fading, represents an alternative explanation for at least part of the observed change. Other potential influences include observer bias, photographic bias, adherence variability, and unmeasured confounding factors. Because this was a single uncontrolled case, it is also not possible to determine which component, if any, of the multimodal regimen contributed to the observed outcome.

During the documented one-month observation period, no treatment-related adverse effects or complications were reported. However, the short duration of follow-up and absence of a standardized adverse event assessment preclude conclusions regarding long-term safety, tolerability, recurrence, rebound hyperpigmentation, hypopigmentation, or delayed adverse reactions. Statements regarding additional improvement with continued treatment or prevention of recurrence cannot be supported by the available follow-up and have therefore been avoided.

Photoprotection is an important consideration in the management of post-inflammatory hyperpigmentation because ultraviolet and visible light exposure may contribute to persistent or recurrent pigmentation. However, sufficiently detailed documentation regarding the specific photoprotection regimen and adherence was not available in this case; therefore, its contribution to the observed clinical course cannot be independently assessed.

This report has several important limitations. These include the single patient design; absence of an untreated control or comparator; the possibility of spontaneous improvement as part of the natural evolution of post inflammatory hyperpigmentation; lack of objective pigmentation measurements and validated scar assessment scales; non standardized photographic assessment; potential observer and photographic bias; short-term follow up; incomplete characterization of the proprietary treatment formulation; inability to determine which component of the multimodal regimen, if any, contributed to the observed change; incomplete documentation of adherence and potential concomitant treatments; and other unmeasured confounding factors. Additional prospective studies using standardized treatment protocols, objective pigmentation measurements, appropriate comparator groups, standardized photography, and longer follow-up are needed before conclusions regarding efficacy, safety, reproducibility, or comparative effectiveness can be established.

## Conclusions

This case report describes visually observed clinical improvement in post-inflammatory hyperpigmentation following treatment with the NewMelan depigmentation protocol. A reduction in pigmentation intensity and improvement in skin tone uniformity were observed on clinical examination and serial photographic assessment at approximately one month of follow-up. However, objective pigmentation measurements, standardized photographic assessment, and an untreated control or comparator were not available. Therefore, treatment efficacy and causality cannot be established from this single uncontrolled case, and spontaneous improvement cannot be excluded as a potential explanation for the observed change. Further studies using objective outcome measures, standardized photography, comparator groups, and longer follow-up are needed to evaluate the efficacy, safety, and reproducibility of depigmentation protocols for post-burn post-inflammatory hyperpigmentation.
